# Supplementary material for: Distributed compact plasma reactor decontamination for planetary protection in space missions
Source: Sci Rep. 2023 Feb 2;13:1928. doi: 10.1038/s41598-023-29049-2 (PMC9894852; doi:10.1038/s41598-023-29049-2)
Supplement: Supplementary file 1 — Supplementary Tables. [file 41598_2023_29049_MOESM1_ESM.docx]

**Supplementary Data for the manuscript: Distributed Compact Plasma Reactor Sterilization for Planetary Protection in Space Missions**

**Bhaswati Choudhury^1,2^, Tamara Revazishvili^2^, Maria Lozada^1^, Sarthak Roy^1^, Emma Noelle Mastro^1,2^, Sherlie Portugal^1,3^, Subrata Roy^1,4^**

^1^SurfPlasma, Inc, 32601, Gainesville, USA.

^2^Emerging Pathogens Institute, University of Florida, Gainesville, Fl-32611, USA.

^3^School of Electrical Engineering, Technological University of Panama, Panama City, Panama.

^4^Dept. of Mechanical and Aerospace Engineering, University of Florida, Gainesville, Fl-32611, USA.

*** Correspondence:**Subrata Roy, subrata@surfplasma.com

**Keywords: plasma, planetary protection, ozone, flow actuation, sterilization, dielectric barrier discharge.**

**Supplementary Data:**

This document contains supplementary data for the manuscript titled ‘Distributed Compact Plasma Reactor Sterilization for Planetary Protection in Space Missions’.

**Table S1**: **Summary of sterilization experiments with *E.coli***

| **Test Organism: *E. coli*** | | | | | | |
| --- | --- | --- | --- | --- | --- | --- |
| **Expt no.** | **Coupon Material** | **No. of CPPR** | **Exposure time (mins)**  **[CPPR on and off alternately]** | **Control**  **Log _10_ (CFU/coupon)** | **Log reduction averaged over 11 points in the chamber**  **Log _10_ (CFU/coupon)** | **Complete killing** |
| 1 | Aluminum | 4 | 5 [5+ 0] | 4.5 | 0.26+0.07 | No |
| 2 | Aluminum | 3 | 10 [10 + 0] | 4.6 | 0.53+0.15 | No |
| 3 | Aluminum | 4 | 15 [10+5] | 4.8 | 1.87+0.41 | No |
| 4 | Aluminum | 4 | 20 [15+5] | 4.8 | 4.8+0.00 | **Yes** |
| 5 | Aluminum | 4 | 20 [15+5] | 4.4 | 4.4+0.00 | **Yes** |
| 6 | Aluminum | 4 | 30 [15+5+5+5] | 4.6 | 4.6+0.00 | **Yes** |
| 7 | Orthofabric | 4 | 15 [10+5] | 4.1 | 4.1+0.00 | **Yes** |
| 8 | Orthofabric | 4 | 30 [25+5] | 4.1 | 4.1+0.00 | **Yes** |
| 9 | Aluminum | 4 | 20 [15+5] | 5.2 | 5.2+0.00 | **Yes** |
| 10 | Aluminum | 4 | 20 [15+5] | 5.4 | 5.4+0.00 | **Yes** |
| 11 | Polycarbonate | 4 | 20 [15+5] | 5.6 | 5.6+0.00 | **Yes** |
| 12 | Polycarbonate | 4 | 20 [15+5] | 5.7 | 5.7+0.00 | **Yes** |
| 13 | Orthofabric | 4 | 20 [15+5] | 4.9 | 4.9+0.00 | **Yes** |
| 14 | Orthofabric | 4 | 20 [15+5] | 5.3 | 5.3+0.00 | **Yes** |
| 15 | Kevlar | 4 | 20 [15+5] | 4.7 | 4.7+0.00 | **Yes** |
| 16 | Kevlar | 4 | 20 [15+5] | 4 | 4+0.00 | **Yes** |
| 17 | Orthofabric | 4 | 20 [15+5] | 3.6 | 3.6+0.00 | **Yes** |
| 18 | Kevlar | 4 | 20 [15+5] | 4.9 | 4.9+0.00 | **Yes** |
| 19 | Polycarbonate | 4 | 20 [15+5] | 5.3 | 5.3+0.00 | **Yes** |

**Table S2**: **Summary of all experiments with *B. subtilis***

| **Test Organism: *B. subtilis*** | | | | | | |
| --- | --- | --- | --- | --- | --- | --- |
| **Expt no.** | **Coupon Material** | **No. of CPPR** | **Exposure time (mins)**  **[CPPR on and off alternately]** | **Control**  **Log _10_ (CFU/coupon)** | **Log reduction averaged over 11 points in the chamber**  **Log _10_ (CFU/coupon)** | **Complete killing** |
| 20 | Polycarbonate | 3 | 10 [10+0] | 5.1 | 0.76+0.15 | No |
| 21 | Polycarbonate | 4 | 20 [15+5] | 5.2 | 1.31+0.09 | No |
| 22 | Polycarbonate | 4 | 30 [15+5+5+5] | 5.6 | 1.40+0.29 | No |
| 23 | Polycarbonate | 4 | 30 [15+5+5+5] | 5.1 | 1.38+0.20 | No |
| 24 | Aluminum | 4 | 25 [20+5] | 4.6 | 1.6+0.14 | No |
| 25 | Kevlar | 6 | 30 [25+5] | 4 | 3.5+0.8 | No |
| 26 | Kevlar | 6 | 30 [25+5] | 3.6 | 3+0.8 | No |
| 27 | Orthofabric | 6 | 30 [25+5] | 3.3 | 3.3+0 | **Yes** |
| 28 | Aluminum | 6 | 30 [25+5] | 4.4 | 4.2+1 | No |
| 29 | Polycarbonate | 6 | 30 [25+5] | 5 | 5 | **Yes** |
| 30 | Aluminum | 6 | 30 [25+5] | 4.5 | 4.5 | **Yes** |
| 31 | Orthofabric | 6 | 30 [25+5] | 3.6 | 3.6 | **Yes** |
| 32 | Kevlar | 6 | 30 [25+5] | 4.3 | 4.3 | **Yes** |
| 33 | Polycarbonate | 6 | 30 [25+5] | 4.4 | 4.4 | **Yes** |
| 34 | Aluminum | 6 | 30 [25+5] | 5 | 5 | **Yes** |
| 35 | Orthofabric | 6 | 30 [25+5] | 3.7 | 3.7 | **Yes** |
| 36 | Orthofabric | 6 | 30 [25+5] | 4.4 | 4.4 | **Yes** |
| 37 | Polycarbonate | 6 | 30 [25+5] | 5.2 | 5.2 | **Yes** |

**Experiment 1:**

Test organism: *E. Coli*

Coupon material: Aluminum

No. of CPPRs used: 4

Exposure time: 5 minutes (CPPRs on throughout)

Table S3. Distribution in disinfection in the internal volume of the APS prototype

| **Control** | **Exposed** | | | **Log reduction** |
| --- | --- | --- | --- | --- |
| **CFU/coupon** | **Plane** | **Position** | **CFU/coupon** | **Log _10_ (CFU/coupon)** |
| 3.05E+04 | P1 | Q3R3 | 1.61E+04 | 0.27 |
|  | P2 | Q1R1 | 1.67E+04 | 0.26 |
|  |  | Q3R1 | 1.62E+04 | 0.27 |
|  |  | Q5R1 | 1.62E+04 | 0.27 |
|  |  | Q1R3 | 1.25E+04 | 0.38 |
|  |  | Q3R3 | 1.67E+04 | 0.26 |
|  |  | Q5R3 | 2.25E+04 | 0.13 |
|  |  | Q1R5 | 1.67E+04 | 0.26 |
|  |  | Q3R5 | 1.59E+04 | 0.28 |
|  |  | Q5R5 | 1.46E+04 | 0.32 |
|  | P3 | Q3R3 | 2.21E+04 | 0.14 |
| **Average log reduction inside chamber** | | | | **0.26+0.07** |

**Experiment 2:**

Test organism: *E. Coli*

Coupon material: Aluminum

No. of CPPRs used: 3

Exposure time: 10 minutes (CPPRs on throughout)

Table S4. Distribution in disinfection in the internal volume of the APS prototype

| **Control** | **Exposed** | | | **Log reduction** |
| --- | --- | --- | --- | --- |
| **CFU/coupon** | **Plane** | **Position** | **CFU/coupon** | **Log _10_ (CFU/coupon)** |
| 3.70E+04 | P1 | Q3R3 | 7.80E+03 | 0.67 |
|  | P2 | Q1R1 | 6.30E+03 | 0.76 |
|  |  | Q3R1 | 2.03E+04 | 0.25 |
|  |  | Q5R1 | 8.55E+03 | 0.63 |
|  |  | Q1R3 | 1.32E+04 | 0.44 |
|  |  | Q3R3 | 1.25E+04 | 0.46 |
|  |  | Q5R3 | 9.15E+03 | 0.60 |
|  |  | Q1R5 | 1.31E+04 | 0.44 |
|  |  | Q3R5 | 7.65E+03 | 0.68 |
|  |  | Q5R5 | 1.41E+04 | 0.41 |
|  | P3 | Q3R3 | 1.50E+03 | 1.38 |
| **Average log reduction inside chamber** | | | | **0.53+0.15** |

**Experiment 3:**

Test organism: *E. Coli*

Coupon material: Aluminum

No. of CPPRs used: 4

Exposure time: 15 minutes (10 minutes CPPR on + 5 minutes CPPR off)

Table S5. Distribution in disinfection in the internal volume of the APS prototype

| **Control** | **Exposed** | | | **Log reduction** |
| --- | --- | --- | --- | --- |
| **CFU/coupon** | **Plane** | **Position** | **CFU/coupon** | **Log _10_ (CFU/coupon)** |
| 3.20E+04 | P1 | Q3R3 | 3.15E+02 | 2.00 |
|  | P2 | Q1R1 | 5.85E+02 | 1.73 |
|  |  | Q3R1 | 5.70E+02 | 1.74 |
|  |  | Q5R1 | 2.55E+02 | 2.09 |
|  |  | Q1R3 | 6.75E+02 | 1.67 |
|  |  | Q3R3 | 2.40E+03 | 1.12 |
|  |  | Q5R3 | 6.00E+02 | 1.72 |
|  |  | Q1R5 | 4.50E+01 | 2.85 |
|  |  | Q3R5 | 3.15E+02 | 2.00 |
|  |  | Q5R5 | 5.55E+02 | 1.76 |
|  | P3 | Q3R3 | 3.45E+02 | 1.96 |
| **Average log reduction inside chamber** | | | | **1.87+0.41** |

**Experiment 4:**

Test organism: *E. Coli*

Coupon material: Aluminum

No. of CPPRs used: 4

Exposure time: 20 minutes (15 minutes CPPR on + 5 minutes CPPR off)

Table S6. Distribution in disinfection in the internal volume of the APS prototype

| **Control** | **Exposed** | | | **Log reduction** |
| --- | --- | --- | --- | --- |
| **CFU/coupon** | **Plane** | **Position** | **CFU/coupon** | **Log _10_ (CFU/coupon)** |
| 5.80E+04 | P1 | Q3R3 | 0 | 4.8 |
|  | P2 | Q1R1 | 0 | 4.8 |
|  |  | Q3R1 | 0 | 4.8 |
|  |  | Q5R1 | 0 | 4.8 |
|  |  | Q1R3 | 0 | 4.8 |
|  |  | Q3R3 | 0 | 4.8 |
|  |  | Q5R3 | 0 | 4.8 |
|  |  | Q1R5 | 0 | 4.8 |
|  |  | Q3R5 | 0 | 4.8 |
|  |  | Q5R5 | 0 | 4.8 |
|  | P3 | Q3R3 | 0 | 4.8 |
| **Average log reduction inside chamber** | | | | **4.8+0.00** |

**Experiment 5:**

Test organism: *E. Coli*

Coupon material: Aluminum

No. of CPPRs used: 4

Exposure time: 20 minutes (15 minutes CPPR on + 5 minutes CPPR off)

Table S7. Distribution in disinfection in the internal volume of the APS prototype

| **Control** | **Exposed** | | | **Log reduction** |
| --- | --- | --- | --- | --- |
| **CFU/coupon** | **Plane** | **Position** | **CFU/coupon** | **Log _10_ (CFU/coupon)** |
| 2.35E+04 | P1 | Q3R3 | 0 | 4.4 |
|  | P2 | Q1R1 | 0 | 4.4 |
|  |  | Q3R1 | 0 | 4.4 |
|  |  | Q5R1 | 0 | 4.4 |
|  |  | Q1R3 | 0 | 4.4 |
|  |  | Q3R3 | 0 | 4.4 |
|  |  | Q5R3 | 0 | 4.4 |
|  |  | Q1R5 | 0 | 4.4 |
|  |  | Q3R5 | 0 | 4.4 |
|  |  | Q5R5 | 0 | 4.4 |
|  | P3 | Q3R3 | 0 | 4.4 |
| **Average log reduction inside chamber** | | | | **4.4+0.00** |

**Experiment 6:**

Test organism: *E. Coli*

Coupon material: Aluminum

No. of CPPRs used: 4

Exposure time: 30 minutes (15 minutes CPPR on + 5 minutes CPPR off +5 minutes CPPR on + 5 minutes CPPR off)

Table S8. Distribution in disinfection in the internal volume of the APS prototype

| **Control** | **Exposed** | | | **Log reduction** |
| --- | --- | --- | --- | --- |
| **CFU/coupon** | **Plane** | **Position** | **CFU/coupon** | **Log _10_ (CFU/coupon)** |
| 3.85E+04 | P1 | Q3R3 | 0 | 4.6 |
|  | P2 | Q1R1 | 0 | 4.6 |
|  |  | Q3R1 | 0 | 4.6 |
|  |  | Q5R1 | 0 | 4.6 |
|  |  | Q1R3 | 0 | 4.6 |
|  |  | Q3R3 | 0 | 4.6 |
|  |  | Q5R3 | 0 | 4.6 |
|  |  | Q1R5 | 0 | 4.6 |
|  |  | Q3R5 | 0 | 4.6 |
|  |  | Q5R5 | 0 | 4.6 |
|  | P3 | Q3R3 | 0 | 4.6 |
| **Average log reduction inside chamber** | | | | **4.6+0.00** |

**Experiment 7:**

Test organism: *E. Coli*

Coupon material: Orthofabric

No. of CPPRs used: 4

Exposure time: 15 minutes (10 minutes CPPR on + 5 minutes CPPR off)

Table S9. Distribution in disinfection in the internal volume of the APS prototype

| **Control** | **Exposed** | | | **Log reduction** |
| --- | --- | --- | --- | --- |
| **CFU/coupon** | **Plane** | **Position** | **CFU/coupon** | **Log _10_ (CFU/coupon)** |
| 1.00E+04 | P1 | Q3R3 | 0 | 4.1 |
|  | P2 | Q1R1 | 0 | 4.1 |
|  |  | Q3R1 | 0 | 4.1 |
|  |  | Q5R1 | 0 | 4.1 |
|  |  | Q1R3 | 0 | 4.1 |
|  |  | Q3R3 | 0 | 4.1 |
|  |  | Q5R3 | 0 | 4.1 |
|  |  | Q1R5 | 0 | 4.1 |
|  |  | Q3R5 | 0 | 4.1 |
|  |  | Q5R5 | 0 | 4.1 |
|  | P3 | Q3R3 | 0 | 4.1 |
| **Average log reduction inside chamber** | | | | **4.1+0.00** |

**Experiment 8:**

Test organism: *E. Coli*

Coupon material: Orthofabric

No. of CPPRs used: 4

Exposure time: 30 minutes (25 minutes CPPR on + 5 minutes CPPR off)

Table S10. Distribution in disinfection in the internal volume of the APS prototype

| **Control** | **Exposed** | | | **Log reduction** |
| --- | --- | --- | --- | --- |
| **CFU/coupon** | **Plane** | **Position** | **CFU/coupon** | **Log _10_ (CFU/coupon)** |
| 1.00E+04 | P1 | Q3R3 | 0 | 4.1 |
|  | P2 | Q1R1 | 0 | 4.1 |
|  |  | Q3R1 | 0 | 4.1 |
|  |  | Q5R1 | 0 | 4.1 |
|  |  | Q1R3 | 0 | 4.1 |
|  |  | Q3R3 | 0 | 4.1 |
|  |  | Q5R3 | 0 | 4.1 |
|  |  | Q1R5 | 0 | 4.1 |
|  |  | Q3R5 | 0 | 4.1 |
|  |  | Q5R5 | 0 | 4.1 |
|  | P3 | Q3R3 | 0 | 4.1 |
| **Average log reduction inside chamber** | | | | **4.1+0.00** |

**Experiment 9:**

Test organism: *E. Coli*

Coupon material: Aluminum

No. of CPPRs used: 4

Exposure time: 20 minutes (15 minutes CPPR on + 5 minutes CPPR off)

Table S11. Distribution in disinfection in the internal volume of the APS prototype

| **Control** | **Exposed** | | | **Log reduction** |
| --- | --- | --- | --- | --- |
| **CFU/coupon** | **Plane** | **Position** | **CFU/coupon** | **Log _10_ (CFU/coupon)** |
| 1.58E+05 | P1 | Q3R3 | 0 | 5.2 |
|  | P2 | Q1R1 | 0 | 5.2 |
|  |  | Q3R1 | 0 | 5.2 |
|  |  | Q5R1 | 0 | 5.2 |
|  |  | Q1R3 | 0 | 5.2 |
|  |  | Q3R3 | 0 | 5.2 |
|  |  | Q5R3 | 0 | 5.2 |
|  |  | Q1R5 | 0 | 5.2 |
|  |  | Q3R5 | 0 | 5.2 |
|  |  | Q5R5 | 0 | 5.2 |
|  | P3 | Q3R3 | 0 | 5.2 |
| **Average log reduction inside chamber** | | | | **5.2+0.00** |

**Experiment 10:**

Test organism: *E. Coli*

Coupon material: Aluminum

No. of CPPRs used: 4

Exposure time: 20 minutes (15 minutes CPPR on + 5 minutes CPPR off)

Table S12. Distribution in disinfection in the internal volume of the APS prototype

| **Control** | **Exposed** | | | **Log reduction** |
| --- | --- | --- | --- | --- |
| **CFU/coupon** | **Plane** | **Position** | **CFU/coupon** | **Log _10_ (CFU/coupon)** |
| 2.51E+05 | P1 | Q3R3 | 0 | 5.4 |
|  | P2 | Q1R1 | 0 | 5.4 |
|  |  | Q3R1 | 0 | 5.4 |
|  |  | Q5R1 | 0 | 5.4 |
|  |  | Q1R3 | 0 | 5.4 |
|  |  | Q3R3 | 0 | 5.4 |
|  |  | Q5R3 | 0 | 5.4 |
|  |  | Q1R5 | 0 | 5.4 |
|  |  | Q3R5 | 0 | 5.4 |
|  |  | Q5R5 | 0 | 5.4 |
|  | P3 | Q3R3 | 0 | 5.4 |
| **Average log reduction inside chamber** | | | | **5.4+0.00** |

**Experiment 11:**

Test organism: *E. Coli*

Coupon material: Polycarbonate

No. of CPPRs used: 4

Exposure time: 20 minutes (15 minutes CPPR on + 5 minutes CPPR off)

Table S13. Distribution in disinfection in the internal volume of the APS prototype

| **Control** | **Exposed** | | | **Log reduction** |
| --- | --- | --- | --- | --- |
| **CFU/coupon** | **Plane** | **Position** | **CFU/coupon** | **Log _10_ (CFU/coupon)** |
| 3.98E+05 | P1 | Q3R3 | 0 | 5.6 |
|  | P2 | Q1R1 | 0 | 5.6 |
|  |  | Q3R1 | 0 | 5.6 |
|  |  | Q5R1 | 0 | 5.6 |
|  |  | Q1R3 | 0 | 5.6 |
|  |  | Q3R3 | 0 | 5.6 |
|  |  | Q5R3 | 0 | 5.6 |
|  |  | Q1R5 | 0 | 5.6 |
|  |  | Q3R5 | 0 | 5.6 |
|  |  | Q5R5 | 0 | 5.6 |
|  | P3 | Q3R3 | 0 | 5.6 |
| **Average log reduction inside chamber** | | | | **5.6+0.00** |

**Experiment 12:**

Test organism: *E. Coli*

Coupon material: Polycarbonate

No. of CPPRs used: 4

Exposure time: 20 minutes (15 minutes CPPR on + 5 minutes CPPR off)

Table S14. Distribution in disinfection in the internal volume of the APS prototype

| **Control** | **Exposed** | | | **Log reduction** |
| --- | --- | --- | --- | --- |
| **CFU/coupon** | **Plane** | **Position** | **CFU/coupon** | **Log _10_ (CFU/coupon)** |
| 5.01E+05 | P1 | Q3R3 | 0 | 5.7 |
|  | P2 | Q1R1 | 0 | 5.7 |
|  |  | Q3R1 | 0 | 5.7 |
|  |  | Q5R1 | 0 | 5.7 |
|  |  | Q1R3 | 0 | 5.7 |
|  |  | Q3R3 | 0 | 5.7 |
|  |  | Q5R3 | 0 | 5.7 |
|  |  | Q1R5 | 0 | 5.7 |
|  |  | Q3R5 | 0 | 5.7 |
|  |  | Q5R5 | 0 | 5.7 |
|  | P3 | Q3R3 | 0 | 5.7 |
| **Average log reduction inside chamber** | | | | **5.7+0.00** |

**Experiment 13:**

Test organism: *E. Coli*

Coupon material: Orthofabric

No. of CPPRs used: 4

Exposure time: 20 minutes (15 minutes CPPR on + 5 minutes CPPR off)

Table S15. Distribution in disinfection in the internal volume of the APS prototype

| **Control** | **Exposed** | | | **Log reduction** |
| --- | --- | --- | --- | --- |
| **CFU/coupon** | **Plane** | **Position** | **CFU/coupon** | **Log _10_ (CFU/coupon)** |
| 7.94E+04 | P1 | Q3R3 | 0 | 4.9 |
|  | P2 | Q1R1 | 0 | 4.9 |
|  |  | Q3R1 | 0 | 4.9 |
|  |  | Q5R1 | 0 | 4.9 |
|  |  | Q1R3 | 0 | 4.9 |
|  |  | Q3R3 | 0 | 4.9 |
|  |  | Q5R3 | 0 | 4.9 |
|  |  | Q1R5 | 0 | 4.9 |
|  |  | Q3R5 | 0 | 4.9 |
|  |  | Q5R5 | 0 | 4.9 |
|  | P3 | Q3R3 | 0 | 4.9 |
| **Average log reduction inside chamber** | | | | **4.9+0.00** |

**Experiment 14:**

Test organism: *E. Coli*

Coupon material: Orthofabric

No. of CPPRs used: 4

Exposure time: 20 minutes (15 minutes CPPR on + 5 minutes CPPR off)

Table S16. Distribution in disinfection in the internal volume of the APS prototype

| **Control** | **Exposed** | | | **Log reduction** |
| --- | --- | --- | --- | --- |
| **CFU/coupon** | **Plane** | **Position** | **CFU/coupon** | **Log _10_ (CFU/coupon)** |
| 2.00E+05 | P1 | Q3R3 | 0 | 5.3 |
|  | P2 | Q1R1 | 0 | 5.3 |
|  |  | Q3R1 | 0 | 5.3 |
|  |  | Q5R1 | 0 | 5.3 |
|  |  | Q1R3 | 0 | 5.3 |
|  |  | Q3R3 | 0 | 5.3 |
|  |  | Q5R3 | 0 | 5.3 |
|  |  | Q1R5 | 0 | 5.3 |
|  |  | Q3R5 | 0 | 5.3 |
|  |  | Q5R5 | 0 | 5.3 |
|  | P3 | Q3R3 | 0 | 5.3 |
| **Average log reduction inside chamber** | | | | **5.3+0.00** |

**Experiment 15:**

Test organism: *E. Coli*

Coupon material: Kevlar

No. of CPPRs used: 4

Exposure time: 20 minutes (15 minutes CPPR on + 5 minutes CPPR off)

Table S17. Distribution in disinfection in the internal volume of the APS prototype

| **Control** | **Exposed** | | | **Log reduction** |
| --- | --- | --- | --- | --- |
| **CFU/coupon** | **Plane** | **Position** | **CFU/coupon** | **Log _10_ (CFU/coupon)** |
| 5.01E+04 | P1 | Q3R3 | 0 | 4.7 |
|  | P2 | Q1R1 | 0 | 4.7 |
|  |  | Q3R1 | 0 | 4.7 |
|  |  | Q5R1 | 0 | 4.7 |
|  |  | Q1R3 | 0 | 4.7 |
|  |  | Q3R3 | 0 | 4.7 |
|  |  | Q5R3 | 0 | 4.7 |
|  |  | Q1R5 | 0 | 4.7 |
|  |  | Q3R5 | 0 | 4.7 |
|  |  | Q5R5 | 0 | 4.7 |
|  | P3 | Q3R3 | 0 | 4.7 |
| **Average log reduction inside chamber** | | | | **4.7+0.00** |

**Experiment 16:**

Test organism: *E. Coli*

Coupon material: Kevlar

No. of CPPRs used: 4

Exposure time: 20 minutes (15 minutes CPPR on + 5 minutes CPPR off)

Table S18. Distribution in disinfection in the internal volume of the APS prototype

| **Control** | **Exposed** | | | **Log reduction** |
| --- | --- | --- | --- | --- |
| **CFU/coupon** | **Plane** | **Position** | **CFU/coupon** | **Log _10_ (CFU/coupon)** |
| 1.00E+04 | P1 | Q3R3 | 0 | 4 |
|  | P2 | Q1R1 | 0 | 4 |
|  |  | Q3R1 | 0 | 4 |
|  |  | Q5R1 | 0 | 4 |
|  |  | Q1R3 | 0 | 4 |
|  |  | Q3R3 | 0 | 4 |
|  |  | Q5R3 | 0 | 4 |
|  |  | Q1R5 | 0 | 4 |
|  |  | Q3R5 | 0 | 4 |
|  |  | Q5R5 | 0 | 4 |
|  | P3 | Q3R3 | 0 | 4 |
| **Average log reduction inside chamber** | | | | **4.0+0.00** |

**Experiment 17:**

Test organism: *E. Coli*

Coupon material: Orthofabric

No. of CPPRs used: 4

Exposure time: 20 minutes (15 minutes CPPR on + 5 minutes CPPR off)

Table S19. Distribution in disinfection in the internal volume of the APS prototype

| **Control** | **Exposed** | | | **Log reduction** |
| --- | --- | --- | --- | --- |
| **CFU/coupon** | **Plane** | **Position** | **CFU/coupon** | **Log _10_ (CFU/coupon)** |
| 3.98E+03 | P1 | Q3R3 | 0 | 3.6 |
|  | P2 | Q1R1 | 0 | 3.6 |
|  |  | Q3R1 | 0 | 3.6 |
|  |  | Q5R1 | 0 | 3.6 |
|  |  | Q1R3 | 0 | 3.6 |
|  |  | Q3R3 | 0 | 3.6 |
|  |  | Q5R3 | 0 | 3.6 |
|  |  | Q1R5 | 0 | 3.6 |
|  |  | Q3R5 | 0 | 3.6 |
|  |  | Q5R5 | 0 | 3.6 |
|  | P3 | Q3R3 | 0 | 3.6 |
| **Average log reduction inside chamber** | | | | **3.6+0.00** |

**Experiment 18:**

Test organism: *E. Coli*

Coupon material: Kevlar

No. of CPPRs used: 4

Exposure time: 20 minutes (15 minutes CPPR on + 5 minutes CPPR off)

Table S20. Distribution in disinfection in the internal volume of the APS prototype

| **Control** | **Exposed** | | | **Log reduction** |
| --- | --- | --- | --- | --- |
| **CFU/coupon** | **Plane** | **Position** | **CFU/coupon** | **Log _10_ (CFU/coupon)** |
| 7.94E+04 | P1 | Q3R3 | 0 | 4.9 |
|  | P2 | Q1R1 | 0 | 4.9 |
|  |  | Q3R1 | 0 | 4.9 |
|  |  | Q5R1 | 0 | 4.9 |
|  |  | Q1R3 | 0 | 4.9 |
|  |  | Q3R3 | 0 | 4.9 |
|  |  | Q5R3 | 0 | 4.9 |
|  |  | Q1R5 | 0 | 4.9 |
|  |  | Q3R5 | 0 | 4.9 |
|  |  | Q5R5 | 0 | 4.9 |
|  | P3 | Q3R3 | 0 | 4.9 |
| **Average log reduction inside chamber** | | | | **4.9+0.00** |

**Experiment 19:**

Test organism: *E. Coli*

Coupon material: Polycarbonate

No. of CPPRs used: 4

Exposure time: 20 minutes (15 minutes CPPR on + 5 minutes CPPR off)

Table S21. Distribution in disinfection in the internal volume of the APS prototype

| **Control** | **Exposed** | | | **Log reduction** |
| --- | --- | --- | --- | --- |
| **CFU/coupon** | **Plane** | **Position** | **CFU/coupon** | **Log _10_ (CFU/coupon)** |
| 2.00E+05 | P1 | Q3R3 | 0 | 5.3 |
|  | P2 | Q1R1 | 0 | 5.3 |
|  |  | Q3R1 | 0 | 5.3 |
|  |  | Q5R1 | 0 | 5.3 |
|  |  | Q1R3 | 0 | 5.3 |
|  |  | Q3R3 | 0 | 5.3 |
|  |  | Q5R3 | 0 | 5.3 |
|  |  | Q1R5 | 0 | 5.3 |
|  |  | Q3R5 | 0 | 5.3 |
|  |  | Q5R5 | 0 | 5.3 |
|  | P3 | Q3R3 | 0 | 5.3 |
| **Average log reduction inside chamber** | | | | **5.3+0.00** |

**Experiment 20:**

Test organism: *B.Subtilis*

Coupon material: Polycarbonate

No. of CPPRs used: 3

Exposure time: 10 minutes (CPPRs on throughout)

Table S22. Distribution in disinfection in the internal volume of the APS prototype

| **Control** | **Exposed** | | | **Log reduction** |
| --- | --- | --- | --- | --- |
| **CFU/coupon** | **Plane** | **Position** | **CFU/coupon** | **Log _10_ (CFU/coupon)** |
| 1.23E+05 | P1 | Q3R3 | 2.63E+04 | 0.68 |
|  | P2 | Q1R1 | 1.71E+04 | 0.87 |
|  |  | Q3R1 | 2.31E+04 | 0.74 |
|  |  | Q5R1 | 2.46E+04 | 0.71 |
|  |  | Q1R3 | 2.78E+04 | 0.66 |
|  |  | Q3R3 | 2.70E+04 | 0.67 |
|  |  | Q5R3 | 2.09E+04 | 0.78 |
|  |  | Q1R5 | 2.24E+04 | 0.75 |
|  |  | Q3R5 | 2.72E+04 | 0.67 |
|  |  | Q5R5 | 2.79E+04 | 0.65 |
|  | P3 | Q3R3 | 7.95E+03 | 1.20 |
| **Average log reduction inside chamber** | | | | **0.76+0.15** |

**Experiment 21:**

Test organism: *B.Subtilis*

Coupon material: Polycarbonate

No. of CPPRs used: 4

Exposure time: 20 minutes (15 minutes CPPR on + 5 minutes CPPR off)

Table S23. Distribution in disinfection in the internal volume of the APS prototype

| **Control** | **Exposed** | | | **Log reduction** |
| --- | --- | --- | --- | --- |
| **CFU/coupon** | **Plane** | **Position** | **CFU/coupon** | **Log _10_ (CFU/coupon)** |
| 1.61E+05 | P1 | Q3R3 | 9.00E+03 | 1.25 |
|  | P2 | Q1R1 | 6.75E+03 | 1.37 |
|  |  | Q3R1 | 7.20E+03 | 1.34 |
|  |  | Q5R1 | 5.70E+03 | 1.44 |
|  |  | Q1R3 | 1.23E+04 | 1.11 |
|  |  | Q3R3 | 6.15E+03 | 1.41 |
|  |  | Q5R3 | 7.95E+03 | 1.30 |
|  |  | Q1R5 | ** | ** |
|  |  | Q3R5 | 9.30E+03 | 1.23 |
|  |  | Q5R5 | 7.20E+03 | 1.34 |
|  | P3 | Q3R3 | 8.10E+03 | 1.29 |
| **Average log reduction inside chamber** | | | | **1.31+0.09** |

**Experiment 22:**

Test organism: *B.Subtilis*

Coupon material: Polycarbonate

No. of CPPRs used: 4

Exposure time: 30 minutes (15 minutes CPPR on + 5 minutes CPPR off +5 minutes CPPR on + 5 minutes CPPR off)

Table S24. Distribution in disinfection in the internal volume of the APS prototype

| **Control** | **Exposed** | | | **Log reduction** |
| --- | --- | --- | --- | --- |
| **CFU/coupon** | **Plane** | **Position** | **CFU/coupon** | **Log _10_ (CFU/coupon)** |
| 4.75E+05 | P1 | Q3R3 | 3.00E+04 | 1.22 |
|  | P2 | Q1R1 | 2.30E+04 | 1.34 |
|  |  | Q3R1 | 2.12E+04 | 1.37 |
|  |  | Q5R1 | 3.90E+03 | 2.11 |
|  |  | Q1R3 | 2.76E+04 | 1.26 |
|  |  | Q3R3 | 4.50E+03 | 2.05 |
|  |  | Q5R3 | 3.75E+04 | 1.13 |
|  |  | Q1R5 | 3.00E+04 | 1.22 |
|  |  | Q3R5 | 3.00E+04 | 1.22 |
|  |  | Q5R5 | 2.25E+04 | 1.35 |
|  | P3 | Q3R3 | 1.23E+04 | 1.61 |
| **Average log reduction inside chamber** | | | | **1.40+0.29** |

**Experiment 23:**

Test organism: *B.Subtilis*

Coupon material: Polycarbonate

No. of CPPRs used: 4

Exposure time: 30 minutes (15 minutes CPPR on + 5 minutes CPPR off +5 minutes CPPR on + 5 minutes CPPR off)

Table S25. Distribution in disinfection in the internal volume of the APS prototype

| **Control** | **Exposed** | | | **Log reduction** |
| --- | --- | --- | --- | --- |
| **CFU/coupon** | **Plane** | **Position** | **CFU/coupon** | **Log _10_ (CFU/coupon)** |
| 1.24E+05 | P1 | Q3R3 | 3.45E+03 | 1.56 |
|  | P2 | Q1R1 | 3.60E+03 | 1.54 |
|  |  | Q3R1 | 6.30E+03 | 1.30 |
|  |  | Q5R1 | 1.05E+04 | 1.08 |
|  |  | Q1R3 | 4.65E+03 | 1.43 |
|  |  | Q3R3 | 6.00E+02 | 2.32 |
|  |  | Q5R3 | 2.25E+03 | 1.75 |
|  |  | Q1R5 | 6.60E+03 | 1.28 |
|  |  | Q3R5 | 4.50E+03 | 1.45 |
|  |  | Q5R5 | 6.30E+03 | 1.30 |
|  | P3 | Q3R3 | 9.00E+03 | 1.15 |
| **Average log reduction inside chamber** | | | | **1.38+0.20** |

**Experiment 24:**

Test organism: *B.Subtilis*

Coupon material: Aluminum

No. of CPPRs used: 4

Exposure time: 25 minutes (20 minutes CPPR on + 5 minutes CPPR off)

Table S26. Distribution in disinfection in the internal volume of the APS prototype

| **Control** | **Exposed** | | | **Log reduction** |
| --- | --- | --- | --- | --- |
| **CFU/coupon** | **Plane** | **Position** | **CFU/coupon** | **Log _10_ (CFU/coupon)** |
| 1.23E+05 | P1 | Q3R3 | 2.70E+03 | 1.67 |
|  | P2 | Q1R1 | 2.10E+03 | 1.78 |
|  |  | Q3R1 | 2.70E+03 | 1.67 |
|  |  | Q5R1 | 4.80E+03 | 1.42 |
|  |  | Q1R3 | 4.35E+03 | 1.46 |
|  |  | Q3R3 | 4.35E+03 | 1.46 |
|  |  | Q5R3 | 2.40E+03 | 1.72 |
|  |  | Q1R5 | ** | ** |
|  |  | Q3R5 | 2.10E+03 | 1.78 |
|  |  | Q5R5 | 4.65E+03 | 1.43 |
|  | P3 | Q3R3 | 3.30E+03 | 1.58 |
| **Average log reduction inside chamber** | | | | **1.6+0.14** |

**Experiment 25:**

Test organism: *B.Subtilis*

Coupon material: Kevlar

No. of CPPRs used: 6

Exposure time: 30 minutes (25 minutes CPPR on + 5 minutes CPPR off)

Table S27. Distribution in disinfection in the internal volume of the APS prototype

| **Control** | **Exposed** | | | **Log reduction** |
| --- | --- | --- | --- | --- |
| **CFU/coupon** | **Plane** | **Position** | **CFU/coupon** | **Log _10_ (CFU/coupon)** |
| 1.26E+04 | P1 | Q3R3 | 0.00E+00 | 4.1 |
|  | P2 | Q1R1 | 0.00E+00 | 4.1 |
|  |  | Q3R1 | 0.00E+00 | 4.1 |
|  |  | Q5R1 | 0.00E+00 | 4.1 |
|  |  | Q1R3 | 0.00E+00 | 4.1 |
|  |  | Q3R3 | 6.00E+01 | 2.32 |
|  |  | Q5R3 | 1.95E+02 | 1.80 |
|  |  | Q1R5 | 0.00E+00 | 4.1 |
|  |  | Q3R5 | 3.00E+01 | 2.62 |
|  |  | Q5R5 | 0.00E+00 | 4.1 |
|  | P3 | Q3R3 | 0.00E+00 | 4.1 |
| **Average log reduction inside chamber** | | | | **3.5+0.8** |

**Experiment 26:**

Test organism: *B.Subtilis*

Coupon material: Kevlar

No. of CPPRs used: 6

Exposure time: 30 minutes (25 minutes CPPR on + 5 minutes CPPR off)

Table S28. Distribution in disinfection in the internal volume of the APS prototype

| **Control** | **Exposed** | | | **Log reduction** |
| --- | --- | --- | --- | --- |
| **CFU/coupon** | **Plane** | **Position** | **CFU/coupon** | **Log _10_ (CFU/coupon)** |
| 4.07E+03 | P1 | Q3R3 | 0.00E+00 | 3.6 |
|  | P2 | Q1R1 | 0.00E+00 | 3.6 |
|  |  | Q3R1 | 0.00E+00 | 3.6 |
|  |  | Q5R1 | 0.00E+00 | 3.6 |
|  |  | Q1R3 | 3.00E+01 | 2.12 |
|  |  | Q3R3 | 6.00E+01 | 1.82 |
|  |  | Q5R3 | 7.50E+01 | 1.72 |
|  |  | Q1R5 | 0.00E+00 | 3.6 |
|  |  | Q3R5 | 4.50E+01 | 1.94 |
|  |  | Q5R5 | 0.00E+00 | 3.6 |
|  | P3 | Q3R3 | 0.00E+00 | 3.6 |
| **Average log reduction inside chamber** | | | | **3.0+0.8** |

**Experiment 27:**

Test organism: *B.Subtilis*

Coupon material: Orthofabric

No. of CPPRs used: 6

Exposure time: 30 minutes (25 minutes CPPR on + 5 minutes CPPR off)

Table S29. Distribution in disinfection in the internal volume of the APS prototype

| **Control** | **Exposed** | | | **Log reduction** |
| --- | --- | --- | --- | --- |
| **CFU/coupon** | **Plane** | **Position** | **CFU/coupon** | **Log _10_ (CFU/coupon)** |
| 2.00E+03 | P1 | Q3R3 | 0 | 3.3 |
|  | P2 | Q1R1 | 0 | 3.3 |
|  |  | Q3R1 | 0 | 3.3 |
|  |  | Q5R1 | 0 | 3.3 |
|  |  | Q1R3 | 0 | 3.3 |
|  |  | Q3R3 | 0 | 3.3 |
|  |  | Q5R3 | 0 | 3.3 |
|  |  | Q1R5 | 0 | 3.3 |
|  |  | Q3R5 | 0 | 3.3 |
|  |  | Q5R5 | 0 | 3.3 |
|  | P3 | Q3R3 | 0 | 3.3 |
| **Average log reduction inside chamber** | | | | **3.3+0** |

**Experiment 28:**

Test organism: *B.Subtilis*

Coupon material: Aluminum

No. of CPPRs used: 6

Exposure time: 30 minutes (25 minutes CPPR on + 5 minutes CPPR off)

Table S30. Distribution in disinfection in the internal volume of the APS prototype

| **Control** | **Exposed** | | | **Log reduction** |
| --- | --- | --- | --- | --- |
| **CFU/coupon** | **Plane** | **Position** | **CFU/coupon** | **Log _10_ (CFU/coupon)** |
| 2.51E+04 | P1 | Q3R3 | 0.00E+00 | 4.4 |
|  | P2 | Q1R1 | 1.50E+01 | 3.22 |
|  |  | Q3R1 | 0.00E+00 | 4.4 |
|  |  | Q5R1 | 0.00E+00 | 4.4 |
|  |  | Q1R3 | 0.00E+00 | 4.4 |
|  |  | Q3R3 | 0.00E+00 | 4.4 |
|  |  | Q5R3 | 0.00E+00 | 4.4 |
|  |  | Q1R5 | 0.00E+00 | 4.4 |
|  |  | Q3R5 | 1.50E+01 | 3.22 |
|  |  | Q5R5 | 0.00E+00 | 4.4 |
|  | P3 | Q3R3 | 0.00E+00 | 4.4 |
| **Average log reduction inside chamber** | | | | **4.2+1** |

**Experiment 29:**

Test organism: *B.Subtilis*

Coupon material: Polycarbonate

No. of CPPRs used: 6

Exposure time: 30 minutes (25 minutes CPPR on + 5 minutes CPPR off)

Table S31. Distribution in disinfection in the internal volume of the APS prototype

| **Control** | **Exposed** | | | **Log reduction** |
| --- | --- | --- | --- | --- |
| **CFU/coupon** | **Plane** | **Position** | **CFU/coupon** | **Log _10_ (CFU/coupon)** |
| 1.00E+05 | P1 | Q3R3 | 0 | 5 |
|  | P2 | Q1R1 | 0 | 5 |
|  |  | Q3R1 | 0 | 5 |
|  |  | Q5R1 | 0 | 5 |
|  |  | Q1R3 | 0 | 5 |
|  |  | Q3R3 | 0 | 5 |
|  |  | Q5R3 | 0 | 5 |
|  |  | Q1R5 | 0 | 5 |
|  |  | Q3R5 | 0 | 5 |
|  |  | Q5R5 | 0 | 5 |
|  | P3 | Q3R3 | 0 | 5 |
| **Average log reduction inside chamber** | | | | **5+0** |

**Experiment 30:**

Test organism: *B.Subtilis*

Coupon material: Polycarbonate

No. of CPPRs used: 6

Exposure time: 30 minutes (25 minutes CPPR on + 5 minutes CPPR off)

Table S32. Distribution in disinfection in the internal volume of the APS prototype

| **Control** | **Exposed** | | | **Log reduction** |
| --- | --- | --- | --- | --- |
| **CFU/coupon** | **Plane** | **Position** | **CFU/coupon** | **Log _10_ (CFU/coupon)** |
| 3.16E+04 | P1 | Q3R3 | 0 | 4.5 |
|  | P2 | Q1R1 | 0 | 4.5 |
|  |  | Q3R1 | 0 | 4.5 |
|  |  | Q5R1 | 0 | 4.5 |
|  |  | Q1R3 | 0 | 4.5 |
|  |  | Q3R3 | 0 | 4.5 |
|  |  | Q5R3 | 0 | 4.5 |
|  |  | Q1R5 | 0 | 4.5 |
|  |  | Q3R5 | 0 | 4.5 |
|  |  | Q5R5 | 0 | 4.5 |
|  | P3 | Q3R3 | 0 | 4.5 |
| **Average log reduction inside chamber** | | | | **4.5+0** |

**Experiment 31:**

Test organism: *B.Subtilis*

Coupon material: Orthofabric

No. of CPPRs used: 6

Exposure time: 30 minutes (25 minutes CPPR on + 5 minutes CPPR off)

Table S33. Distribution in disinfection in the internal volume of the APS prototype

| **Control** | **Exposed** | | | **Log reduction** |
| --- | --- | --- | --- | --- |
| **CFU/coupon** | **Plane** | **Position** | **CFU/coupon** | **Log _10_ (CFU/coupon)** |
| 3.98E+03 | P1 | Q3R3 | 0 | 3.6 |
|  | P2 | Q1R1 | 0 | 3.6 |
|  |  | Q3R1 | 0 | 3.6 |
|  |  | Q5R1 | 0 | 3.6 |
|  |  | Q1R3 | 0 | 3.6 |
|  |  | Q3R3 | 0 | 3.6 |
|  |  | Q5R3 | 0 | 3.6 |
|  |  | Q1R5 | 0 | 3.6 |
|  |  | Q3R5 | 0 | 3.6 |
|  |  | Q5R5 | 0 | 3.6 |
|  | P3 | Q3R3 | 0 | 3.6 |
| **Average log reduction inside chamber** | | | | **3.6+0** |

**Experiment 32:**

Test organism: *B.Subtilis*

Coupon material: Kevlar

No. of CPPRs used: 6

Exposure time: 30 minutes (25 minutes CPPR on + 5 minutes CPPR off)

Table S34. Distribution in disinfection in the internal volume of the APS prototype

| **Control** | **Exposed** | | | **Log reduction** |
| --- | --- | --- | --- | --- |
| **CFU/coupon** | **Plane** | **Position** | **CFU/coupon** | **Log _10_ (CFU/coupon)** |
| 2.00E+04 | P1 | Q3R3 | 0 | 4.3 |
|  | P2 | Q1R1 | 0 | 4.3 |
|  |  | Q3R1 | 0 | 4.3 |
|  |  | Q5R1 | 0 | 4.3 |
|  |  | Q1R3 | 0 | 4.3 |
|  |  | Q3R3 | 0 | 4.3 |
|  |  | Q5R3 | 0 | 4.3 |
|  |  | Q1R5 | 0 | 4.3 |
|  |  | Q3R5 | 0 | 4.3 |
|  |  | Q5R5 | 0 | 4.3 |
|  | P3 | Q3R3 | 0 | 4.3 |
| **Average log reduction inside chamber** | | | | **4.3+0** |

**Experiment 33:**

Test organism: *B.Subtilis*

Coupon material: Polycarbonate

No. of CPPRs used: 6

Exposure time: 30 minutes (25 minutes CPPR on + 5 minutes CPPR off)

Table S35. Distribution in disinfection in the internal volume of the APS prototype

| **Control** | **Exposed** | | | **Log reduction** |
| --- | --- | --- | --- | --- |
| **CFU/coupon** | **Plane** | **Position** | **CFU/coupon** | **Log _10_ (CFU/coupon)** |
| 2.51E+04 | P1 | Q3R3 | 0 | 4.4 |
|  | P2 | Q1R1 | 0 | 4.4 |
|  |  | Q3R1 | 0 | 4.4 |
|  |  | Q5R1 | 0 | 4.4 |
|  |  | Q1R3 | 0 | 4.4 |
|  |  | Q3R3 | 0 | 4.4 |
|  |  | Q5R3 | 0 | 4.4 |
|  |  | Q1R5 | 0 | 4.4 |
|  |  | Q3R5 | 0 | 4.4 |
|  |  | Q5R5 | 0 | 4.4 |
|  | P3 | Q3R3 | 0 | 4.4 |
| **Average log reduction inside chamber** | | | | **4.4+0** |

**Experiment 34:**

Test organism: *B.Subtilis*

Coupon material: Aluminum

No. of CPPRs used: 6

Exposure time: 30 minutes (25 minutes CPPR on + 5 minutes CPPR off)

Table S36. Distribution in disinfection in the internal volume of the APS prototype

| **Control** | **Exposed** | | | **Log reduction** |
| --- | --- | --- | --- | --- |
| **CFU/coupon** | **Plane** | **Position** | **CFU/coupon** | **Log _10_ (CFU/coupon)** |
| 1.00E+05 | P1 | Q3R3 | 0 | 5 |
|  | P2 | Q1R1 | 0 | 5 |
|  |  | Q3R1 | 0 | 5 |
|  |  | Q5R1 | 0 | 5 |
|  |  | Q1R3 | 0 | 5 |
|  |  | Q3R3 | 0 | 5 |
|  |  | Q5R3 | 0 | 5 |
|  |  | Q1R5 | 0 | 5 |
|  |  | Q3R5 | 0 | 5 |
|  |  | Q5R5 | 0 | 5 |
|  | P3 | Q3R3 | 0 | 5 |
| **Average log reduction inside chamber** | | | | **5+0** |

**Experiment 35:**

Test organism: *B.Subtilis*

Coupon material: Orthofabric

No. of CPPRs used: 6

Exposure time: 30 minutes (25 minutes CPPR on + 5 minutes CPPR off)

Table S37. Distribution in disinfection in the internal volume of the APS prototype

| **Control** | **Exposed** | | | **Log reduction** |
| --- | --- | --- | --- | --- |
| **CFU/coupon** | **Plane** | **Position** | **CFU/coupon** | **Log _10_ (CFU/coupon)** |
| 5.01E+03 | P1 | Q3R3 | 0 | 3.7 |
|  | P2 | Q1R1 | 0 | 3.7 |
|  |  | Q3R1 | 0 | 3.7 |
|  |  | Q5R1 | 0 | 3.7 |
|  |  | Q1R3 | 0 | 3.7 |
|  |  | Q3R3 | 0 | 3.7 |
|  |  | Q5R3 | 0 | 3.7 |
|  |  | Q1R5 | 0 | 3.7 |
|  |  | Q3R5 | 0 | 3.7 |
|  |  | Q5R5 | 0 | 3.7 |
|  | P3 | Q3R3 | 0 | 3.7 |
| **Average log reduction inside chamber** | | | | **3.7+0** |

**Experiment 36:**

Test organism: *B.Subtilis*

Coupon material: Orthofabric

No. of CPPRs used: 6

Exposure time: 30 minutes (25 minutes CPPR on + 5 minutes CPPR off)

Table S38. Distribution in disinfection in the internal volume of the APS prototype

| **Control** | **Exposed** | | | **Log reduction** |
| --- | --- | --- | --- | --- |
| **CFU/coupon** | **Plane** | **Position** | **CFU/coupon** | **Log _10_ (CFU/coupon)** |
| 2.51E+04 | P1 | Q3R3 | 0 | 4.4 |
|  | P2 | Q1R1 | 0 | 4.4 |
|  |  | Q3R1 | 0 | 4.4 |
|  |  | Q5R1 | 0 | 4.4 |
|  |  | Q1R3 | 0 | 4.4 |
|  |  | Q3R3 | 0 | 4.4 |
|  |  | Q5R3 | 0 | 4.4 |
|  |  | Q1R5 | 0 | 4.4 |
|  |  | Q3R5 | 0 | 4.4 |
|  |  | Q5R5 | 0 | 4.4 |
|  | P3 | Q3R3 | 0 | 4.4 |
| **Average log reduction inside chamber** | | | | **4.4+0** |

**Experiment 37:**

Test organism: *B.Subtilis*

Coupon material: Polycarbonate

No. of CPPRs used: 6

Exposure time: 30 minutes (25 minutes CPPR on + 5 minutes CPPR off)

Table S39. Distribution in disinfection in the internal volume of the APS prototype

| **Control** | **Exposed** | | | **Log reduction** |
| --- | --- | --- | --- | --- |
| **CFU/coupon** | **Plane** | **Position** | **CFU/coupon** | **Log _10_ (CFU/coupon)** |
| 1.58E+05 | P1 | Q3R3 | 0 | 5.2 |
|  | P2 | Q1R1 | 0 | 5.2 |
|  |  | Q3R1 | 0 | 5.2 |
|  |  | Q5R1 | 0 | 5.2 |
|  |  | Q1R3 | 0 | 5.2 |
|  |  | Q3R3 | 0 | 5.2 |
|  |  | Q5R3 | 0 | 5.2 |
|  |  | Q1R5 | 0 | 5.2 |
|  |  | Q3R5 | 0 | 5.2 |
|  |  | Q5R5 | 0 | 5.2 |
|  | P3 | Q3R3 | 0 | 5.2 |
| **Average log reduction inside chamber** | | | | **5.2+0** |
